# Non-invasive photoacoustic computed tomography of rat heart anatomy and function

**DOI:** 10.1038/s41377-022-01053-7

**Published:** 2023-01-03

**Authors:** Li Lin, Xin Tong, Susana Cavallero, Yide Zhang, Shuai Na, Rui Cao, Tzung K. Hsiai, Lihong V. Wang

**Affiliations:** 1 Caltech Optical Imaging Laboratory, Andrew and Peggy Cherng Department of Medical Engineering, Department of Electrical Engineering, California Institute of Technology, Pasadena, CA USA; 2 Department of Bioengineering, UCLA, Los Angeles, CA USA; 3 Division of Cardiology, Department of Medicine, UCLA, Los Angeles, CA USA; 4 Present Address: College of Biomedical Engineering and Instrument Science, Zhejiang University, Hangzhou, China; 5 Present Address: The First Affiliated Hospital, Zhejiang University School of Medicine, Hangzhou, China

**Keywords:** Imaging and sensing, Photoacoustics

## Abstract

Complementary to mainstream cardiac imaging modalities for preclinical research, photoacoustic computed tomography (PACT) can provide functional optical contrast with high imaging speed and resolution. However, PACT has not been demonstrated to reveal the dynamics of whole cardiac anatomy or vascular system without surgical procedure (thoracotomy) for tissue penetration. Here, we achieved non-invasive imaging of rat hearts using the recently developed three-dimensional PACT (3D-PACT) platform, demonstrating the regulated illumination and detection schemes to reduce the effects of optical attenuation and acoustic distortion through the chest wall; thereby, enabling unimpeded visualization of the cardiac anatomy and intracardiac hemodynamics following rapidly scanning the heart within 10 s. We further applied 3D-PACT to reveal distinct cardiac structural and functional changes among the healthy, hypertensive, and obese rats, with optical contrast to uncover differences in cardiac chamber size, wall thickness, and hemodynamics. Accordingly, 3D-PACT provides high imaging speed and nonionizing penetration to capture the whole heart for diagnosing the animal models, holding promises for clinical translation to cardiac imaging of human neonates.

## Abstract (web summary)

High-speed non-invasive photoacoustic tomography reveals cardiac anatomy and cardiovascular hemodynamics in rats.

## Graphical abstract

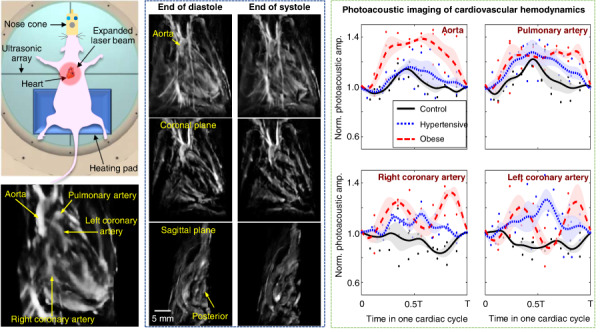

## Introduction

Cardiovascular disease remains the leading cause of morbidity, responsible for 16% of the world’s total deaths^1^. Diagnostic imaging and therapeutic targets warrant preclinical investigations of animal models^2,3^ to understand the cardiac disease mechanisms. Over the last several decades, non-invasive imaging of small animal models provides in vivo insights into the structural and functional phenotypes with physiological and clinical relevance. The current non-invasive imaging modalities allow from a wide range of vertebrate animal models, including light-sheet fluorescent microscopy^4^, echocardiography^5^, magnetic resonance imaging (MRI)^6^, x-ray computed tomography (CT)^7^, positron emission tomography (PET)^8^, and/or single-photon emission computed tomography (SPECT)^9^, and a combination of these imaging techniques^10–12^. Each imaging technique provides the unique degree of tissue penetration, resolution, and contrast to image the specific animal models from zebrafish to mouse to swine models, and the combination of these complementary techniques allows for addressing the spatial and temporal resolution, field of view (FOV), and relative phenotypes in response to the particular imaging needs. For example, echocardiography is a portable tool to assess cardiac function by interrogating the contracting/relaxing heart chambers and open/closure of the valves in real-time. In contrast, CT and MRI are bulky but offer larger FOV and/or finer spatial resolution needed for cardiac anatomy and vascular system^7,13^.

Complementary to the well-established imaging modalities, photoacoustic (PA) computed tomography (PACT) is an emerging imaging technique that combines functional optical contrast from light illumination and high spatial resolution from acoustic detection. Compared with echocardiography, PACT offers rich optical contrasts that are physiologically relevant^14^ and suffers a lower level of speckling artifacts^15^. As compared with MRI, PACT usually provides a higher imaging speed and a more portable imaging platform. Unlike X-ray CT, PACT uses nonionizing illumination to image cardiac vasculature, avoiding injection of contrast agents. Like PET, PACT can reveal molecular information^16^ with finer spatial resolution obviating the need for ionizing radiation.

Despite these unique advantages, the majority of the published PACT prototypes have not been demonstrated for non-invasive cardiac imaging of the detailed anatomical or functional phenotypes due to suboptimal illumination and detection schemes. This limitation has been recognized by the previous PACT studies, including in vivo imaging of mouse hearts with suboptimal clarity for anatomical/functional details^17,18^ and ex vivo imaging of excised/perfused heart with no intracardiac flow dynamics^19,20^. There are 3 challenges for in vivo cardiac PACT. (1) Ribs and lungs surrounding the heart partially block and disturb PA signals. The same limitation exists in echocardiography; however, PACT only suffers from one-way acoustic disturbance since the PA signals are generated in hearts. (2) The high concentration of hemoglobin and myoglobin renders the myocardium highly absorptive to light. This property may produce high PA signal amplitudes; however, it reduces optical penetration into the heart. (3) The periodic heartbeat requires real-time imaging or motion-correction mechanisms to give the imager’s spatial resolution full play for dynamic cardiac structures. For example, the time-gating is widely used in CT^21^, MRI^22^, and PET^23^, dividing every cardiac cycle into multiple phases and collating all the data from a specific phase for imaging reconstruction.

In this context, we seek to overcome these challenges and enhance PACT for pre-clinical and physiological applications. Here, we modified a recently developed three-dimensional PACT (3D-PACT) platform^24^ with regulated illumination and detection schemes to image the whole rat heart in vivo. This 3D approach allows for a hemispherical detection mesh with an extensive view aperture, detecting PA signals from the heart semi-panoramically to alleviate the acoustic blockage caused by ribs and lungs. In addition, the 1064-nm light suffers less scattering in the biological tissue^25^ than the wavelengths that are commonly used by PACT within the near-infrared window (680–950 nm). During the scanning, we synchronized the PACT measurement with cardiac cycles by gating with the electrocardiogram (ECG). The 3D-PACT scanned the rat heart in 10 s and reconstructed a series of cardiac images based on a time-gating strategy.

The 3D-PACT along with the ECG-aided synchronization largely resolves the challenges from the previous PACT approaches and reveals the whole rat heart anatomy non-invasively. Specifically, the 3D-PACT significantly enhances the image clarity and captures the dynamic changes in the cardiac structure between obese and control (i.e., lean) rats; namely, chamber size, myocardial wall thickness, and intracardiac flow. Moreover, the 3D-PACT is capable of recording the distinct blood flow in various sizes of the vascular system from healthy, obese, and hypertensive rats, including the aortas, pulmonary arteries, left and right coronary arteries. Notably, the 3D-PACT shows the near-term potential to provide cardiac image quality comparable to that of MRI^26^, delivering optical contrast for cardiac structure and function, and providing physiological parameters for blood flow.

## Results

### 3D-PACT synchronization with ECG-gating for 4D (3D space + time domain) non-invasive cardiac imaging

**Fig. 1:**
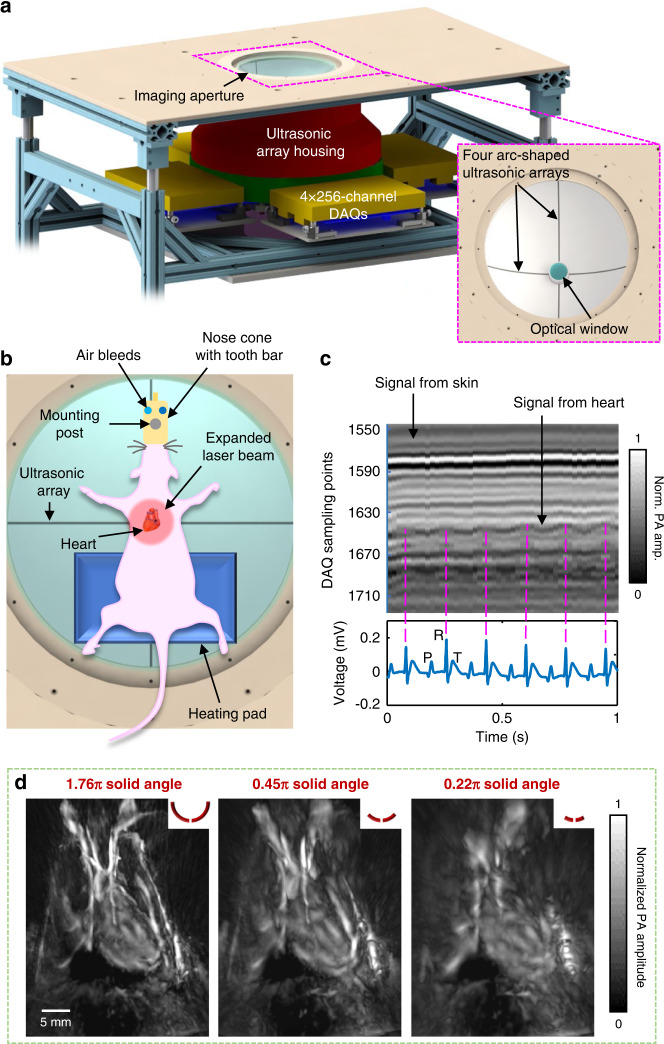
3D-PACT synchronized with ECG for cardiac imaging. **a** Representative sketch of the 3D-PACT platform with a close-up view of the imaging aperture. **b** Experimental setup for the rat heart imaging. The rat was mounted in a prone position, and the expanded light beam was directed towards the rat’s chest from the bottom. **c** Example of the signal synchronization between the 3D-PACT (top) and ECG (bottom) measurements. The 3D-PACT signal was collected from one transducer element on one array at multiple scanning positions (i.e., on the same latitude). In the photoacoustic signal diagram, the horizontal axis represents the scanning steps (i.e., time), and the vertical axis stands for the time-of-flight signal (data acquisition sampling at 20 MHz). The P, R, and T waves in the ECG signals correspond to atrial contraction, ventricular contraction, and ventricular relaxation, respectively. **d** The rat heart images reconstructed with different ultrasonic apertures, showing the impact of a sizeable ultrasonic detection aperture on image reconstruction. The red arcs on the top right corner of each image represent the cross-sectional view of the ultrasonic detection aperture

In the 3D-PACT platform (Fig. 1a), we applied light illumination with a pulse repetition rate of 50 Hz to the subject through an optical window (Figs. 1a and S1). Four arc-shaped ultrasonic transducer arrays rotated coaxially around the optical window for 90° to form a hemispherical detection matrix (Fig. S2 and Methods section). We covered the imaging aperture with a transparent membrane to separate the imaging system from the subject (rat), which was placed in a prone position over the imaging aperture with chest close to the center (Fig. 1b and Methods section). During the 10-s imaging session, the arrays were scanned for 500 steps (see Methods section), and the heart was periodically beating at about 5 Hz^27^. Due to the motion artifacts induced by the heart beats, the reconstructed heart image was largely blurred without motion correction (Fig. S3). Therefore, we applied time-gated motion correction guided by the synchronized ECG measurement (see Methods). For example, to reconstruct the heart image when ventricles are at maximum dilation (end of the ventricular diastole), we only used the photoacoustic data acquired at the scanning positions gated to the R waves of the ECG signals (Fig. 1c). Thus, the 3D approach reveals the time-dependent changes in cardiac structure, including the left vs. right ventricle, free wall vs. septum, pulmonary artery vs. aortic arch, and left vs. right carotid arteries.

### 3D-PACT interrogation of rat cardiac anatomy and vascular system

**Fig. 2:**
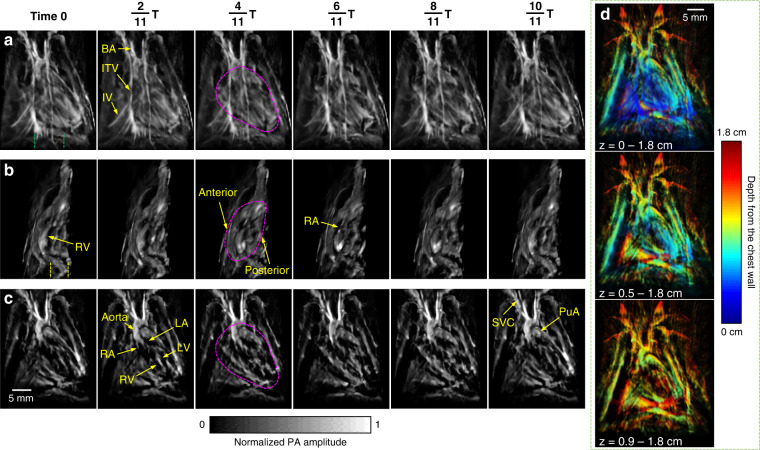
Rat heart anatomy acquired by the 3D-PACT. **a** Front view of the heart within a cardiac cycle. The heart is identified by a magenta circle at $\frac{4}{{11}}{{{\mathrm{T}}}}$. BA, brachiocephalic artery; ITV, internal thoracic vessels; IV, intercostal vessels. **b** Cross-sectional images of the heart on the sagittal plane. Each image is a maximum amplitude projection (MAP) of a slice marked by the green dashed lines in (**a**) (Time 0). **c** Cross-sectional images of the heart on the coronal plane. Each image is an MAP of a slice marked by the yellow dashed lines in (**b**) (Time 0). LA, left atrium; LV, left ventricle; PuA, pulmonary artery; RA, right atrium; RV, right ventricle; SVC, superior vena cava. **d** The same data (at Time 0) shown with color-encoded depths. Shallower structures were peeled away in the lower images to show the posterior anatomy

To address acoustic distortion from the chest ribs and lung air sacs that encompass the heart, we demonstrate a large acoustic detection aperture to collect PA signals from numerous view angles (Fig. 1d). To further improve the PACT reconstruction performance of cardiac anatomy, we synchronized the 3D-PACT with ECG, scanning the heart with ~11 steps (i.e., 11 laser pulses) during each cardiac cycle. Accordingly, we divided every cardiac cycle T into 11 phases and reconstructed a volumetric image for each phase (Fig. 2a and Supplementary Movie 1). Since the image of Phase 1 (i.e., Time 0) was reconstructed using the data acquired at the R waves of the ECG signals, the heart was close to the start of systole. During the $\frac{1}{{11}}{{{\mathrm{T}}}}--\frac{6}{{11}}{{{\mathrm{T}}}}$ period, the heart was in systole but started to relax from $\frac{6}{{11}}{{{\mathrm{T}}}}$. Such a heartbeat pattern complies with the Wiggers diagram. We further mapped the fluctuations of PA signals across the heart (Fig. S4) to take in the heartbeat as dynamic contrast, showing more spatial movements in the apex of the heart than in the top regions.

To reveal the internal cardiac structure, we further showed the cross-sectional images on the sagittal (Fig. 2b and Supplementary Movie 2) and coronal planes (Fig. 2c and Supplementary Movie 3). In the cross-sectional images, we can identify the dynamic changes in atria and ventricles during cardiac contraction (systole) and relaxation (diastole). In Fig. 2c, the end ventricular diastolic volume was observed near Time 0, whereas the end atrial diastolic volume was near $\frac{6}{{11}}{{{\mathrm{T}}}}$. We further encoded the imaging depth by colors and peeled away the structures from the chest wall towards the posterior region of the heart, showing cardiac structures at different depths (Fig. 2d). The imaging contrast of the cardiac anatomy acquired by the 3D-PACT is from optical absorption of myoglobin from the myocardium and hemoglobin from the blood^28^.

### 3D-PACT interrogation of hypertrophic hearts in the obese rats

It is widely recognized that overweight and obesity are cardiometabolic risk factors for developing acute coronary syndromes and stroke^29^. Obesity-associated increase in cholesterol levels and vascular inflammation are recognized to cause ventricular remodeling and to reduce blood perfusion to the myocardium^30^. Here, we demonstrate to capacity of 3D-PACT to interrogate obesity-induced cardiac enlargement or known as hypertrophic abnormalities in cardiac anatomy and function by non-invasively imaging Zucker obese/lean rats of a similar age (Table S1). Compared with the lean rats, whose cardiac cycles typically have 11 phases (11 phases/50 Hz = 0.22 s, i.e., 4.55-Hz cardiac rate), the three obese rats we imaged have 9 phases for each cardiac cycle (i.e., 5.55-Hz cardiac rate), indicating a higher cardiac rate for augmented cardiac output to compensate for the increased metabolic requirements^31^.

**Fig. 3:**
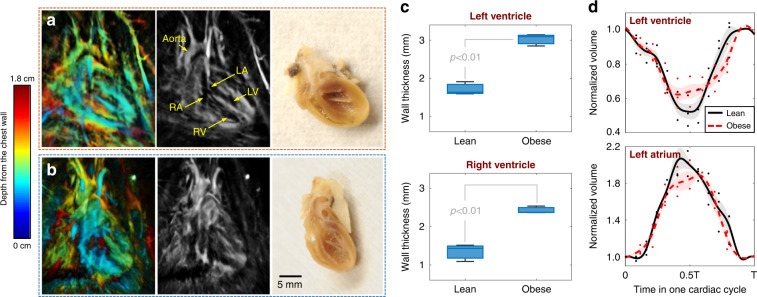
Differences in cardiac anatomy and function between the Zucker obese and lean rats. **a** Cardiac anatomy of a Zucker obese rat. Left panel: Color-encoded depth-resolved image of the heart; middle panel: Cross-sectional image of the heart; Right: Longitudinal sectioning of the corresponding hypertrophic heart. LA, left atrium; LV, left ventricle; RA, right atrium; RV, right ventricle. **b** Cardiac anatomy of a Zucker lean rat (control group). **c** Free wall thickness measurements of the left ventricle (top: *p* < 0.01, *n* = 3) and right ventricle (bottom: *p* < 0.01, *n* = 3) between the obese and lean rats. **d** Relative volume variations of the left ventricles and atriums between the obese and lean rats. The shadow behind curves represents the standard deviation across multiple rats (*n* = 3)

The 3D imaging capability enables the measurement of cardiac anatomy and function. By sectioning the volumetric images on the coronal plane (Fig. 3a, b), we observed concentric hypertrophy^32^ in the obese rat’s heart with increased free wall thicknesses in both left and right ventricles compared to the lean rat (Fig. 3c). For thickness measurement, we selected the middle slice of the heart on the coronal plane and quantified the full-width-at-half-maximum of the photoacoustic signals across the wall section with a length of about 1.3 mm near the middle (Fig. S5). Ex vivo measurements were performed by splitting the dissected hearts on the coronal plane near the middle after imaging, showing concordance with the in vivo measurements (Fig. S6).

In addition, measurements of the changes in cardiac volumes enabled us to assess both the systolic and diastolic function. We divided each heart image into ten sections with specific thicknesses (1.04 mm) on the coronal plane and segmented ventricular and atrial regions to evaluate the volume variations (Fig. S5). To reduce the individual difference within each group (*n* = 3), we normalized the volumes of ventricles and atria to their values at Phase 1 (at the end of diastole). The relative changes in the cardiac function between the lean and obese rats (Figs. 3d and S7) were characterized in terms of the end-systolic ventricular volume and end-diastolic atrial volume of the cardiac cycle. Probably due to the reduced strain of ventricles or well-recognized diastolic dysfunction in the hypertrophic hearts^32^, we observed slightly lower changes in the average of normalized left ventricular volumes in obese rats. Similarly, the left atria in the obese rats developed slightly lower variations in the normalized volume.

### 3D-PACT interrogation of cardiovascular hemodynamics

**Fig. 4:**
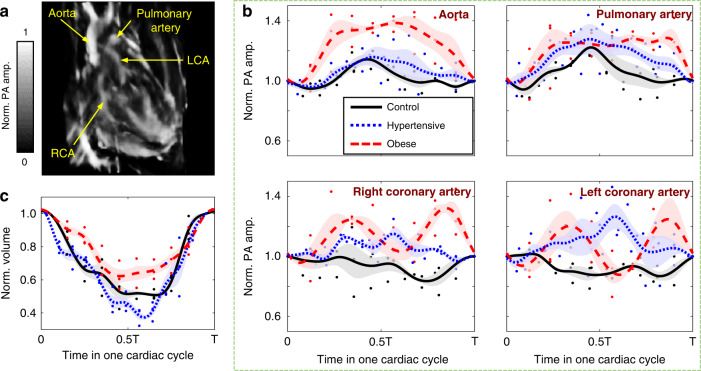
Cardiac hemodynamics in control (i.e., healthy), hypertensive, and obese hearts. **a** A heart image acquired by the 3D-PACT shows the aorta, pulmonary artery, right coronary artery (RCA), and left coronary artery (LCA). **b** PA signal fluctuations in the four cardiac vasculatures among the control, hypertensive, and obese hearts (*n* = 3 for each group). **c** Relative changes in ventricular volume of the control (black solid line), hypertensive (blue dashed line), and hypertrophic (red dashed line) hearts during a cardiac cycle (*n* = 3 for each group)

Given PACT’s high sensitivity to the hematogenous contrast, the 3D-approach enabled periodic hemodynamic interrogation of the cardiovascular system. In a cardiac image acquired by 3D-PACT (Fig. 4a), the aorta, pulmonary artery, right coronary artery, and left coronary artery were captured. To measure the intravascular hemodynamics, we selected a region of interest (ROI) slightly larger than the vessels’ cross-sections and averaged the photoacoustic signal amplitude within a section of 10-voxel length (1.3 mm) along the vessel. Since oxyhemoglobin dominates the optical absorption at 1064 nm (i.e., *μ*_*a* (oxyhemoglobin)_ ≈ 10 × *μ*_*a* (deoxyhemoglobin)_; *μ*_*a*_: absorption coefficient)^33^, the changes in spatially averaged PA signals within the ROI mainly reflect the variations in the amount of oxyhemoglobin, which is determined by the blood volume (or vascular diameter) and oxygen saturation (sO_2_).

The measurements of hemodynamics in the coronary arteries introduce distinct myocardial perfusion among healthy, hypertensive, and obese rats. The averaged PA signals in the aorta and pulmonary artery, peaked during ventricular systole (0.4–0.5 T), showed slightly higher variations in hypertensive hearts (blue dashed lines in Fig. 4b) than in the control group, yet with insignificant statistical differences. This observation could be supported by measuring relative volume changes of the left ventricles (Fig. 4c). In comparison, we noticed more significant fluctuations in the relative PA signal amplitudes from the left coronary arteries in hypertensive hearts within the cardiac cycle (Fig. S8). We speculate that the abnormal hemodynamics are related to the accumulated fat in the damaged arteries, which may affect blood perfusion^34^.

Similarly, we measured and plotted the hemodynamics of cardiac vessels in obese rats. Probably due to the augmented cardiac output and obesity-induced arterial hypertension^35^, we observed higher variations in the averaged PA signals from the four vessels (red dashed lines in Fig. 4b). However, only the right coronary arteries and aortas in the obese rats showed significant statistical differences from the control group (Fig. S8). The combined measurements in cardiac anatomy and hemodynamics depict a potential to provide diagnosis of cardiovascular diseases in individuals with hypertension or obesity.

## Discussion

For decades, the photoacoustic field has faced challenges in cardiac imaging, including acoustic disturbance from ribs and lungs, optical attenuation, and motion artifacts from the periodic heartbeat. Nevertheless, preclinical research and clinical practice are in need of innovative imaging methods with complementary advantages to provide high-dimensional physiological information. In this study, we have demonstrated non-invasive 3D-PACT of cardiac anatomy and function in rats, providing substantial improvements in anatomical image clarity and cardiovascular function measurement. To reveal the cardiac anatomy, 3D-PACT employs a hemispherical acoustic detection aperture with large view angles to mitigate the influences induced by ribs and lungs; The 1064-nm light and uniform illumination also facilitate deep penetration of the entire heart (Fig. 2b); although this study has not provided snapshot or real-time imaging, the periodic heartbeat allows the ECG-guided time-gating method to reduce motion-induced artifacts during a 10-second scan. Clear anatomical imaging further enables measurements of the cardiac functions, including the chamber volume variations and cardiovascular hemodynamics. For example, the changes in photoacoustic signals from the cardiac vessels indicate the variations in blood volume and sO_2_, showing differences in hemodynamics of the hypertensive, hypertrophic, and normal control hearts. In summary, the optical imaging contrast in 3D-PACT depicts the distribution of myoglobin in the myocardium and hemoglobin in the blood, revealing cardiac anatomy and function with physiological relevance^36^.

While the study was performed based on the modification of a recently published imaging system^24^, additional technical innovations are critical to producing clear cardiac images: (1) The ECG-guided time-gating strategy synchronized the 3D-PACT system with the ECG measurement by sending a spike signal to one of the ECG’s electrodes when the system starts to run (see Methods). (2) Regulated laser illumination and gated spatial sampling are synchronized to image the heart dynamics during multiple cardiac cycles (see Methods), providing an FOV large enough for whole-heart imaging in 3D space^37^. (3) In PACT, the light illumination, acoustic detection, and imaging object comprise a comprehensive signal excitation and detection system. Therefore, animal positioning that can reliably match the illumination and detection is usually overlooked but critical for high-quality imaging (see Methods section).

Based on the improvements in this study, PACT of the heart can be further developed towards the following directions: (1) Using ultrasonic arrays with a higher center frequency (e.g., 5–10 MHz) could help reveal detailed cardiac anatomy in small animals with a higher resolution. However, the unwanted effects from acoustic attenuation and rib disturbance will be more significant. (2) To maintain the lateral FOV with an improved imaging speed, rearrangement of the ultrasonic transducers (e.g., eight arc arrays, each with 128 elements) would be implemented to provide an equally dense azimuthal sampling with less scanning steps. (3) The data amplification and acquisition circuits can be modified to integrate ultrasonography, offering well-interpreted imaging contrast (i.e., information) in coregistered images for reference.

3D-PACT is an open imaging platform for preclinical research and clinical translation by simultaneously providing cardiac anatomy and function. Compared with established imaging modalities, PACT of the heart offers additional physiology-related information with high imaging speed and resolution, yet without the need for ionizing radiation or invasiveness, making PACT a promising tool for cardiac imaging of human neonates.

## Materials and methods

### Optical illumination and acoustic detection

For optical illumination, PACT requires a relatively uniform energy deposition within the field of view. While for acoustic detection, high-quality imaging requires a large view angle (i.e., synthetic aperture) and dense sampling in time and space. In 3D-PACT, we directed a 1,064 nm laser beam (DLS9050, Continuum; 50 Hz pulse repetition rate; 5–9 ns pulse width) to the optical window mounted on the scanning axis. The optical window is composed of an engineered diffuser (EDC-15, RPC Photonics Inc.) and a condenser lens (ACL25416U-B, Thorlabs Inc.), expanding the beam to a diameter of ~4.8 cm (Fig. S2). The illumination area is slightly larger than the imaging object (i.e., heart) to lower the requirement of precise animal positioning. Because the heart is filled with blood (i.e., high optical absorption), we used 1064-nm light in this study for less optical attenuation in biological tissues to achieve deep penetration. The optical fluence on the tissue surface (~20 mJ/cm^2^ at 50 Hz) was limited by the American National Standards Institutes’ safety standards^38^.

The acoustic detection module comprises four ultrasonic arc arrays with a separation of 90 degrees. Each array has 256 transducer elements with a central frequency of 2.25 MHz, and each element has a dimension of 0.6 × 0.7 mm^2^, generating a divergence angle (i.e., far-field acoustic diffraction) around 60 degrees covering the imaging object semi-panoramically. Here, we applied a hemispherical detection matrix to enlarge the view angle so the system can acquire acoustic signals propagating in most independent directions. In addition, the 2.25-MHz center frequency of the array also allows the detection of sufficiently low frequency signals that suffer less distortion from the ribs. Notably, this acoustic detection scheme would not avoid distortions from bones and lungs, but would reduce their effects on the reconstructed images. Connecting to the ultrasonic arrays, 1024-channel pre-amplification and analog-to-digital conversion circuits (PhotoSound, Inc.) provide one-to-one mapped data acquisition^24^.

### ECG-guided time gating and spatial sampling

The data acquisition modules and mechanical scanner of the 3D-PACT system were triggered by the laser’s “Lamp Sync Out” trigger output. At the same time, the computer controlled the trigger signal transmission via an electronic relay in the system’s control box. We turned on the laser and ECG systems shortly before closing the relay, which coordinated the data acquisition, laser pulses, and mechanical scanning. We further directed a coaxial cable from the system’s control box to one of the ECG probes (left or right arm) and output a spike signal much higher than the ECG spikes when the relay was closed (Fig. S9). Accordingly, we pinpointed the start time of the photoacoustic data acquisition in the ECG signal, achieving the data synchronization between ECG and 3D-PACT.

We then grouped the PACT data into multiple heartbeat phases based on the synchronized ECG cardiac cycle and reconstructed a volumetric heart image for each phase (Fig. 1c). Since the heart rate was around 5 Hz and the pulsed laser illuminated at 50 Hz, each cardiac cycle can be divided into 9–11 phases, enabling the reconstruction of 9–11 volumetric images to depict the beating heart. The variations in cardiac cycle duration were caused by differences in weight (e.g., obese versus regular rats) and anesthesia (i.e., light versus deep anesthesia). However, the ECG signal shows that the heart rate was relatively stable during the 10-second scan.

For spatial sampling, each of the four arrays was scanned for 500 steps (50 Hz × 10 s) during the 10-second imaging, providing 2000 azimuthal sampling positions. Accordingly, each heart image is reconstructed by ~200 (2000/10 phases) azimuthal positions times 256 polar positions (i.e., each arc array has 256 elements), which could provide an effective FOV with a lateral diameter of 15 mm^37^ and elevational radius of 38 mm.

### Animal preparation

Four strains of rats were imaged in this study (Table S1): (1) Hsd:Sprague Dawley SD rats (120–150 g body weight), (2) Zucker obese rats (200–250 g), (3) Zucker lean rats (120–150 g), (4) Spontaneously hypertensive inbred SHR/NHsd rats (120–150 g). We first imaged the SD rats to optimize the experimental setup and demonstrate the imaging capability (Fig. 2). The Zucker lean rats were imaged as the control group to compare the cardiac anatomy and function with the obese rats (Fig. 3). We compared the cardiovascular hemodynamics in the hypertensive, obese, and SD rats (Fig. 4).

The imaging aperture was covered by a transparent membrane (disposable plastic wrap) that separated the rat from the imaging system and served as a support for the rat. We then covered a feedback-controlled heating pad with a zip bag for waterproofing and placed it above the membrane to maintain the animal’s body temperature (Fig. 1b). Before imaging, we removed the hair on the chest and placed the rat above the heating pad. A 3D-printed nose cone and tooth bar were positioned to provide air with 2% vaporized isoflurane. During imaging, we placed two cross lines above the imaging aperture to mark the center position of the FOV. We further bent the rat’s front limbs towards the back of the body so that the heart would be prominent towards the chest wall. After the imaging session was completed, we euthanized the rat, collected the heart, and obtained a photo of the dissected heart for reference. All the animal experiments followed the protocol approved by the Institutional Animal Care and Use Committee (IACUC) of the California Institute of Technology.

### Image processing and visualization

We used the dual-speed-of-sound back-projection algorithm^39^ implemented in C + + to reconstruct all images with mitigated artifacts induced by the acoustic inhomogeneity between the biological tissue and the coupling fluid (D_2_O). Each volumetric image was reconstructed with a voxel size of 0.13 × 0.13 × 0.13 mm^3^. We then processed each image to improve the contrast through the following steps: (1) A depth compensation (e^0.81×depth (cm)^) method was applied to enhance the PA amplitude in deep tissue. (2) We then used a high-pass filter to suppress the low-frequency background. (3) The high-passed image was further denoised using sparse 4D transform-domain collaborative filtering^40^. (4) To enhance the contrast of blood vessels, a Hessian-based Frangi vesselness filter^41^ was applied to the denoised image. (5) Finally, we added the vesselness-filtered images (self-normalized) with a weighting factor of 0.5 back to the high-passed images (with a weighting factor of 0.5) and obtained the presented images. The color-encoded images in Figs. 2 and 3 were acquired using a specialized 3D visualization software package, namely 3D PA Visualization Studio^42^.

## Supplementary information

Supplementary Information

Supplementary Movie 1

Supplementary Movie 2

Supplementary Movie 3

## Data Availability

All data are available within the Article and Supplementary Files, or available from the authors upon request.
