# Supplementary material for: Non-invasive photoacoustic computed tomography of rat heart anatomy and function
Source: Light Sci Appl. 2023 Jan 3;12:12. doi: 10.1038/s41377-022-01053-7 (PMC9807634; doi:10.1038/s41377-022-01053-7)

**Supplementary Information for Non-invasive Photoacoustic
Computed Tomography of Rat Heart Anatomy and Function**

Li Lin^1,2,3,†^, Xin Tong^1,†^, Susana Cavallero^4^, Yide Zhang^1^, Shuai Na^1^, Rui Cao^1^, Tzung K. Hsiai^4,5,^*, and Lihong V. Wang^1,^*

^1^ Caltech Optical Imaging Laboratory, Andrew and Peggy Cherng Department of Medical Engineering, Department of Electrical Engineering, California Institute of Technology, Pasadena, California, USA.

^2^ Present address 1: College of Biomedical Engineering and Instrument Science, Zhejiang University, Hangzhou, China.

^3^ Present address 2: The First Affiliated Hospital, Zhejiang University School of Medicine, Hangzhou, China.

^4^ Department of Bioengineering, UCLA, Los Angeles, California, USA.

^5^ Division of Cardiology, Department of Medicine, UCLA, Los Angeles, California, USA.

^†^ These authors contributed equally to this work.

* Corresponding author. Email: [THsiai@mednet.ucla.edu](mailto:THsiai@mednet.ucla.edu), [LVW@caltech.edu](mailto:LVW@caltech.edu)

**Supplementary Figures**

**Figure S1. a** Normalized light intensity distribution measured near the tissue surface. **b** Light intensity distribution along the white dashed lines in (a). The beam diameters, defined as the full width at half maximum (FWHM) of the intensity distribution, were around 4.8 cm.

**Figure S2. a** Ultrasonic detection mesh acquired during a 10-second one-way scan, providing 256×2000 sampling positions on the hemispherical surface. **b** Ultrasonic detection mesh for one of the ten heartbeat phases during the scan, generating 256×200 sampling positions on the hemispherical surface.

**Figure S3. a** Front view image of the heart reconstructed without motion correction. ITV, internal thoracic vessels; IV, intercostal vessels. **b** The same heart reconstructed using time-gated motion correction.

**Figure S4. a** Maximum amplitude project of a heart image encoded by the PA-signal fluctuation map. **b** Relative changes of the PA signals from two pixels in (a).

**Figure S5.** Schematic diagrams showing the strategies for measurements of the free wall thickness and chamber volumes in photoacoustic images.

**Figure S6.** Free wall thickness of the ventricles in obese and lean rats (*n* = 3 for each group) acquired by *in vivo* and *ex vivo* measurements.

**Figure S7.** Relative volume changes of right ventricles (left) and atriums (right) in the healthy vs. obese rats within a cardiac cycle (*n* = 3 for each group).

**Figure S8.** Statistical assessment of the range (peak-to-peak value) of photoacoustic signals from specific cardiac vessels in control (i.e., healthy), hypertensive, and obese hearts (*n* = 3 for each group).

**Figure S9.** Synchronization of the ECG measurement with the photoacoustic data acquisition started by sending a spike signal to one of the ECG probes.

**Supplementary Table**

**Table S1.** Characteristics of the rats imaged in this study.


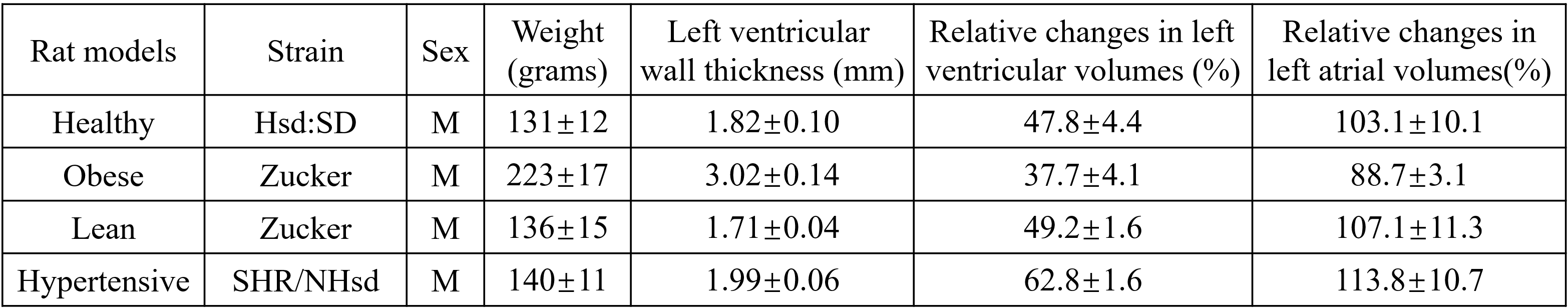

Supplement: Supplementary file 1 — Supplementary Information [file 41377_2022_1053_MOESM1_ESM.docx]
